# Biological Effects of Micro-/Nano-Plastics in Macrophages

**DOI:** 10.3390/nano15050394

**Published:** 2025-03-04

**Authors:** Massimiliano G. Bianchi, Lavinia Casati, Giulia Sauro, Giuseppe Taurino, Erika Griffini, Christian Milani, Marco Ventura, Ovidio Bussolati, Martina Chiu

**Affiliations:** 1 Department of Medicine and Surgery, University of Parma, 43125 Parma, Italy; massimiliano.bianchi@unipr.it (M.G.B.); giuseppe.taurino@unipr.it (G.T.); erika.griffini@unipr.it (E.G.); 2 Microbiome Research Hub, University of Parma, 43125 Parma, Italy; christian.milani@unipr.it (C.M.); marco.ventura@unipr.it (M.V.); 3 Department of Health Sciences, University of Milan, 20122 Milan, Italy; lavinia.casati@unimi.it (L.C.); giulia.sauro@unimi.it (G.S.); 4 Department of Chemistry, Life Sciences and Environmental Sustainability, University of Parma, 43125 Parma, Italy

**Keywords:** inflammation, MAFLD, IBD, innate immunity, macrophages, microbiota, micro-plastics, nano-plastics

## Abstract

The environmental impact of plastics is worsened by their inadequate end-of-life disposal, leading to the ubiquitous presence of micro- (MPs) and nanosized (NPs) plastic particles. MPs and NPs are thus widely present in water and air and inevitably enter the food chain, with inhalation and ingestion as the main exposure routes for humans. Many recent studies have demonstrated that MPs and NPs gain access to several body compartments, where they are taken up by cells, increase the production of reactive oxygen species, and lead to inflammatory changes. In most tissues, resident macrophages engage in the first approach to foreign materials, and this interaction largely affects the subsequent fate of the material and the possible pathological outcomes. On the other hand, macrophages are the main organizers and controllers of both inflammatory responses and tissue repair. Here, we aim to summarize the available information on the interaction of macrophages with MPs and NPs. Particular attention will be devoted to the consequences of this interaction on macrophage viability and functions, as well as to possible implications in pathology.

## 1. Introduction

Micro-plastics (MPs) and nano-plastics (NPs), defined, respectively, as particles with sizes less than 5 mm and 100 nm [1], are now considered an important ecological problem and are causing increasing worries about their effects on human health [2]. The huge quantity of plastic materials produced and their widespread use in a large array of common goods (e.g., water bottles, styrofoam plates, milk bottles, centrifuge tubes, to-go food boxes, plastic bags, tires, disposable masks) has led to an increasing accumulation of debris in the environment and, hence, in the food chain [3]. The recognition of this fact has promoted many studies that have demonstrated the accumulation of plastic micro- and nanomaterials in several tissues of the human body, rendering plausible pathophysiological effects that are still to be fully characterized.

Compared with other widely diffused micromaterials and nanomaterials, investigations on the health effects of MPs and NPs present analogies, significant differences, and open issues. Among the former, nanosized materials have more evident biological effects than microsized materials of the same composition, when directly compared. For instance, Kuai et al. [4] investigated the effects of long-term oral ingestion of MPs/NPs of different sizes on the mouse colon, finding that the nanosized preparation (0.1 μm) had the largest effects in terms of inflammation, alterations in bile acid and carbohydrate metabolism, and inhibition of intestinal motility. More evident toxic effects with smaller MPs/NPs have been observed [5,6]. In the latter contribution, while 0.5 µm MPs entered cells, 5 µm MPs only adhered to the cell membrane, possibly indicating that too large MPs may not be internalized efficiently. However, particles of both sizes induced the generation of reactive oxygen species (ROS).

As for other nanosized materials, when introduced in biological fluids, NPs adsorb protein and other compounds, forming a corona that heavily influences NPs’ fate in the body and interaction with cells, such as macrophages [7]. Macrophages are the most important sentinel cells in human tissues and are among the first cells to contact engineered and natural nanomaterials, largely determining the outcome of the interaction between the body and the material. Remarkably, macrophages internalize a variety of plastic MPs/NPs of different compositions to which humans are increasingly exposed [3], such as polystyrene (PS), polyethylene (PE), polyvinyl chloride (PVC), polypropylene (PP), and polyethylene terephthalate (PET), to name those most frequently encountered in the environment. On the other hand, the advancements in macrophage biology, achieved in the last decade, have led to a more comprehensive understanding of the many roles played by these cells in a variety of diseases.

These facts explain why many studies have been published in the last few years about the effects of MPs and NPs on macrophages and the possible pathophysiological outcomes of this interaction. The aim of this narrative review is to provide an overall, although provisional, status of the art of this rapidly expanding field, and to envisage open issues for further investigations into the biological effects of micro-/nano-plastic on macrophages. Particular attention will be devoted to the outcomes of the interaction on macrophage functions, such as phagocytosis, lysosomal activity, metabolism, polarization, and interaction with microbiota, as well as to possible implications in pathology.

## 2. Cytotoxicity

MPs and NPs are generally endowed with low acute cytotoxicity, and macrophages are not always the most sensitive cell type, at least in vitro. For instance, in the study by Gautam et al. [8], PE MPs are incubated with six different human cell lines derived from tissues representative of potential exposure routes. Only the highest concentration (1000 μg/mL) affected the viability of intestinal epithelial Caco-2 and lung epithelial A549 cells, while no change was detected in the macrophage-like cells evaluated (THP-1 and U937 cell lines). However, in both these macrophage lines, PE MPs increased ROS generation. Interestingly, anti-inflammatory effects were detected in THP-1 and U937 cells, except with THP-1 cells exposed to large-size MPs. However, while mild-to-moderate acute cytotoxicity of MPs and NPs has been most often found, the effects seem dependent on the cell type and, perhaps, on the composition of the particle. Different results may be ascribed to different compositions or to different protein coronas, which may determine the efficiency of engulfment by macrophages or the intracellular effects of the materials, including also the triggering of pyroptosis, a necrotic type of cell death with powerful pro-inflammatory effects [7].

The absence of evident cell death but subtler toxic effects was observed by Tavakolpournegari et al. [9], who used “secondary” MPs/NPs of PET, not synthesized as such but derived from the degradation of plastic goods (see Section 9 for a discussion of the importance of using secondary MPs/NPs in biological tests), to treat mouse alveolar macrophages (MH-S line). No decrease in cell viability was observed after 3 h or 24 h exposure despite efficient internalization. However, ROS increased, mitochondrial membrane potential decreased, and polarization was promoted (see below, Polarization). Also, polyamide MPs lack acute cytotoxic effects [10] and, hence, do not induce acute inflammation. However, sustained exposure of human primary macrophages led to the secretion of interleukin-8 (IL-8/CXCL-8) and activation of the p53 pathway, suggesting the occurrence of genotoxicity. However, although MPs of polytetrafluorethylene (PTFE), which is a material used for nonstick cookware, semiconductors, and medical devices, are endowed with low cytotoxicity, they increase both TNF-α secretion and ROS production, as well as ERK pathway activation in six human cell lines tested, including U937 and THP-1 macrophages [11].

On the contrary, using PS MPs and PS NPs, Rudolph et al. [12] observed toxic effects but only in murine macrophages at the highest dose, and not in epithelial cells. The toxicity of PS MPs/NPs on Raw267.4 macrophages has also been reported by Wang et al., who described cytotoxicity, apoptosis, and pro-inflammatory effects, with the lowest effective concentration (LOEC) much higher for cytotoxicity (1 vs. 0.01 μg/mL) [5]. The molecular mechanisms involved in these toxic effects are beginning to be unveiled [13,14]. PS NPs caused macrophage apoptosis through the cGAS-STING pathway, associated with NF-κB activation, a decrease in mitochondrial membrane potential, metabolic alterations, and the expression of inflammatory genes. *STING* silencing hampered the effects.

Whereas most contributions concern murine macrophages, the interaction of NPs with human macrophages has been directly addressed by Adler et al. [15]. Their experiments demonstrated the uptake of PS MPs and NPs by macrophages derived from the human macrophage-like cell line THP-1 and by primary human macrophages, derived from peripheral blood mononuclear cells (PBMCs). The internalization was demonstrated by transmission electron microscopy, scanning electron microscopy, and live cell imaging. In addition, the reaction of the macrophages was analyzed in terms of metabolic activity (Alamar Blue assay), cytotoxicity (LDH assay), production of ROS, and macrophage polarization. Under the conditions evaluated, NPs increased metabolic activity and cytotoxicity with a dose- and time-dependent effect, while polarization was not substantially affected. The smaller the NPs, the more evident were the effects. PS NPs’ toxic effects on THP-1 cells have also been documented by Kober et al. [16], who detected oxidative stress, decreased mitochondrial membrane potential, and DNA damage.

As for other engineered nanomaterials (nanotubes), surface modification through functionalization significantly affects the interaction with macrophages and its effects. For instance, functionalization with amino groups enhanced the cytotoxicity of PS NPs in Raw264.7 macrophages, compared with either pristine or carboxy-functionalized PS NPs [17].

## 3. Phagocytosis and Intracellular Accumulation

In contrast with epithelial cells of body barriers, macrophages actively engulf MPs and NPs [12]. Fluorescent (Lucifer Yellow) PS NPs have been exploited in that contribution, allowing the demonstration of uptake levels inversely proportional to NPs size at the same dose. Effective phagocytosis of NPs by macrophages, as well as by monocytes and dendritic cells, has been quantified using palladium-doped materials [18]. Effective phagocytosis has also been shown in vivo in murine airway macrophages [19]. Phagocytosis of a proprietary fluorescent MP preparation was associated with cell infiltration, bronchoalveolar macrophage aggregation, increased secretion of TNF-α in bronchoalveolar lavage fluid, and stimulation of Th2-dependent adaptive humoral immunity markers (IgE, IL-4, IL-5) in normal animals, with several alterations exacerbated in asthmatic counterparts. A sizable transcriptomic response followed exposure to MPs in either the control or pathologic model.

In vivo retention and distribution have been investigated with a sophisticated approach by Delaney et al. [20]. Authors functionalized 1 μm and 20 nm amino-functionalized PS particles with isothiocyanate-desferrioxamine (DFO) and radiolabeled them with the positron-emission tomography (PET) tracer [^89^Zr]Zr^4+^. In vitro, the alveolar macrophages MH-S readily internalized the MPs but not the NPs. In vivo, intravenous administration led to the accumulation of both MPs and NPs in the macrophage-rich organs liver, and spleen. After intratracheal instillation, retention of the NPs was much larger than that of the MP preparation.

The biological activities of PS MPs and NPs have been directly correlated with the internalized particles [21]. PS particles ranging from less than 100 nm to 6 μm were not acutely toxic but modified macrophage activities in a size- and dose-dependent manner. Changes in the expression of various surface markers (CD11a/b, CD18, CD86, PD-L1, CD204) were detected, leading the authors to propose that exposure may cause the “emergence of subpopulations of macrophages with an altered phenotype, which may not only be less efficient in their functions, but also alter the fine balance of the innate immune system”.

Among the mechanisms involved in macrophage phagocytosis of MPs/NPs, low-density and high-density lipoprotein receptors (LDLR and SR-B1) have been found to play an essential role in PE-NPs recognition [7]. Kuroiwa et al. [22] identified the T cell immunoglobulin mucin 4 (Tim4), a macrophage receptor for phosphatidylserine on apoptotic cells, as the membrane structure that binds PS MPs. They also propose that the interaction occurs through the extracellular aromatic cluster of the receptor protein, indicating aromatic–aromatic interactions. However, no pro-inflammatory activation was detected upon the interaction, as expected from the anti-inflammatory effects of the interaction between macrophages and apoptotic cells. However, PS MPs blocked efferocytosis and, under in vivo conditions, this effect could promote autoimmune diseases upon chronic exposure.

Once internalized, the intracellular distribution, and perhaps the biological and toxic effects, depend on particle size [23]. Interestingly, neither murine macrophage cell line, exploited in that study, seemed to be endowed with mechanisms for the active excretion of particles. Both nano- and microsized particles were found in the cytoplasm, but only sub-micron particles were detected in the endoplasmic reticulum or the endosomes, possibly yielding a basis for differential toxicity. On the other hand, at least for PS MPs/NPs, no evidence of metabolic degradation has been found, even after the activation of macrophages [24].

As for other nanomaterials, the adsorption of other compounds to the MP/NP surface can powerfully contribute to the biological effects. This issue has been addressed by Yu et al. [25], who investigated the isolated or combined effects of 100 nm PS NPs and 50 μg L^−1^ lead (Pb) on zebrafish intestine with a single-cell approach that allowed the discrimination of the effects on specific cell types. While the combined exposure to 200 μg/L PS NPs and 50 μg/L Pb was the most effective in oxidative stress and inflammation, macrophages exhibited the highest sensitivity to PS NPs with a specific transcriptional response.

Phagocytosis is significantly enhanced by doping the particles with proteins, such as IgG or bovine serum albumin [26]. Proteins adsorbed during the transit in the gastrointestinal tract after oral ingestion may also accelerate MPs/NPs absorption [27]. In that study, the composition of protein corona was investigated using LC–MS/MS, and the uptake of the materials by THP-1 macrophages was determined through flow cytometry and confocal microscopy. Digestion increased the uptake of uncharged MPs smaller than 500 nm but not that of larger or charged MPs. The corona proteins correlated with increased uptake included coagulation factors, apolipoproteins, and vitronectin. The amount and composition of protein corona may be affected by the period spent in the environment by the particle, with aged PS NPs being more hydrophilic and adsorbing more proteins [28]. Also in this case, the adsorbed proteins, derived from the bronchoalveolar fluid (BALF), enhanced the uptake of the NPs by macrophages through clathrin-mediated endocytosis.

## 4. Inability to Digest MPs/NPs, Lysosomal Impairment, Autophagy Defect

Exposure to MPs/NPs can impair lysosomal function and prevent autophagy completion. These effects have been observed also for other undigestible nanomaterials devoid of sizable acute toxicity [29]. In the case of MPs/NPs, the alterations can profoundly affect pathways connected to lipid metabolism in macrophages. Florance et al. [30] report that the exposure to PS NPs (50 μg/mL) led to the formation of lipid-laden foamy cells in Raw264.7 macrophage cultures, with higher doses impairing lysosome function. Lipid accumulation was also observed in PS NPs-exposed BV2 murine microglial cells. Comparable effects were also observed by the same group in human macrophages [31]. Alterations in lipid metabolism in macrophages have also been demonstrated through advanced morphological techniques [32].

These results are of potential interest in defining mechanisms for the atherogenic potential of NPs. Indeed, among the possible detrimental consequences of exposure to these materials, cardiovascular conditions have been recently highlighted by a prospective study on the relationships between exposure and atheroma development [33]. PE was detected in carotid artery plaques of more than 50% of patients, while more than 10% presented detectable amounts of polyvinyl chloride. The mechanisms involved deserve further investigation, although macrophages play a key role in NPs accumulation. In the plaque tissue, NPs were indeed localized close to macrophages, and their presence correlated with a more severe clinical outcome and inflammatory biomarkers. Moreover, a recent contribution [34] demonstrates that exposure to PS NPs stimulates phagocytosis and promotes M1 polarization, indicated by the upregulation of MAcrophage Receptor with COllagenous structure (MARCO). Through MARCO induction, NPs would enhance lipid accumulation and the development of macrophage-derived foam cells. MARCO involvement in the atherogenic potential of PS NPs has also been proposed by Wang et al. [34]. Using a susceptible murine model (ApoE-/- mice, exposed to 2.5–250 mg/kg of PS 50 nm NPs and a high-fat diet for 19 weeks), they found that PS NPs activated phagocytosis of aorta M1 macrophages, induced the MARCO receptor and increased long-chain acyl carnitines (LCACs) through the inhibition of hepatic carnitine palmitoyltransferase 2. These synergistic alterations promoted the formation of foamy cells in the aorta wall plaques.

Impairment of lysosomal function by exposure to PS MPs/NPs has been described in a more stringent real-life scenario by Deng et al. [35], who studied the biological effects of a massive release of MPs and NPs after the exposure of plastic food containers to high temperatures. Murine macrophages promptly engulfed the materials showing a progressive lysosomal impairment. The importance of secondary changes in MPs and NPs surfaces occurring in the environment for the pathogenesis of lysosomal defects has been highlighted by Manabe et al. [36]. These authors compared the effects of PE MPs, either intact or UV-surface-degraded, as a proxy for secondary changes, on Raw264.7 and THP-1 macrophages. Caspase 3-independent cell death was detected after exposure to degraded, but not non-degraded PE MPs. Death was attributed to autophagosome formation, inhibition of autophagy, and lysosomal dysregulation.

## 5. Polarization

Macrophages are extremely plastic cells, able to shift among different functional states, the extreme of which are exemplified by the pro-inflammatory M1 and anti-inflammatory M2 types, characterized by the expression of specific markers [37]. As expected, M1 macrophages show a more active phagocytosis towards PS MPs/NPs than M2 polarized ones or M0 non-polarized cells [23]. In turn, the exposure to MPs and NPs has been demonstrated to drive macrophage polarization, although contradictory results have been described in different models and under experimental conditions.

While Adler et al. found no substantial changes in the polarization of human THP-1 and primary macrophages upon exposure to PS NPs [15], Hu et al. [38] exposed C57BL/6-mated BALB/c mice to the same material and observed a shift in the M1/M2 macrophages ratio to M2 subtype and an increased secretion of immunosuppressive cytokines, together with embryo toxicity. Somewhat comparable results have been reported in primary human macrophages by Wolff et al. [39]. They found that PS and poly methyl methacrylate (PMMA) MPs/NPs downregulated inflammatory M1 markers in polarized macrophages derived from human blood monocytes, leading to M2 macrophage polarization after a 24 h exposure.

However, most of the other studies point to a prevailing M1 polarization of primary murine macrophages and Raw264.7 cells, at least in vitro and for PS MPs/NPs [24,40]. A trend towards M1 polarization has also been repeatedly shown in vivo. The report by Zhao et al. [41] exploits chronic (4 weeks) oral administration, showing that it increased the ratio of natural killer cells and macrophages to non-parenchymal liver cells. The production of typical M1 cytokines (IFN-γ, TNF-α, IL-1β, IL-6, and IL-33) was upregulated (at least at mRNA level), while IL-4, IL-5, IL-10, IL-18, and TGF-β1 were downregulated. The study by Jin et al. also indicates an M1-type activation upon chronic exposure to PS MPs [42].

Interestingly, a time-dependent biphasic pattern was observed by Tavakolpournegari et al. [9], who observed an early (3 h) M1 polarization, followed by the late (24 h) appearance of M2 markers in MH-S murine alveolar macrophages treated with PE NPs. Deranged M2 polarization may explain the defect in tissue repair observed in acetaminophen-induced acute liver damage if animals are pre-treated with PS MPs [43].

## 6. Metabolism

Activation of macrophages is strictly correlated with metabolic changes and, conversely, metabolic features, affecting carbohydrate, lipid, and amino acid metabolism, characterize diverse macrophage polarization.

Alterations of lipid metabolism have been detected with non-targeted metabolomics in Raw264.7 macrophages treated with PS NPs [14]. In particular, decreased production of the anti-inflammatory mediator prostaglandin B1 has been associated with the activation of the STING pathway promoted by the NPs. Altered lipid metabolism occurs in M1-polarized THP-1 macrophages exposed to CdSe-quantum dots-doped PP and PVC NPs [32]. In this case, however, the possibility that Cd-containing quantum dots have biological effects per se should not be excluded [44].

The relationship between micro-plastics and macrophage metabolism has been investigated by Merkley et al. [24]. After phagocytosis of polystyrene MPs, murine macrophages, which are not able to degrade them, shift from oxidative mitochondrial to glycolytic metabolism and increase membrane expression of the co-stimulator proteins CD80 and CD86, two M1 markers, along with cytokine genes. Profound, NPs-dependent modifications in the glycolytic pathway have been described by Li et al. [45]. PS NPs-dependent infiltration of innate immunity cells in testicles is associated with peculiar metabolic changes in the tissue, such as increased 3-phospho-D-glycerate, phosphoenolpyruvate, and lactate concentrations, accompanied by decreased pyruvate and adenosine triphosphate (ATP) production, likely due to downregulated pyruvate kinase M2 activity and an increase in PKM2 dimerization. These changes, along with increased production of pro-inflammatory cytokines and M1 markers, are attributed to NPs-elicited ROS production in macrophages. However, at the tissue level, parenchymal cells are also involved, and PKM2 changes have been documented in testicular parenchymal cells with an elegant co-culture approach [45]. This result implies that exposure of macrophages to NPs can modulate the metabolic features of the whole tissue.

Metabolic changes in immune cells have also been hypothesized after chronic, low-dose exposure to PET NPs based on changes in gene expression in intestinal tissues [46]. In this case, however, the exposure would lead to increased activity of oxidative pathways, which would increase ROS production in the absence of frank inflammation and cytotoxicity.

Defective metabolism of sphingolipids in Raw264.7 cells exposed to PS NPs (0.5 and 5 μm) has been described by Wang et al. [6], possibly attributable to lysosomal impairment (see above, Inability to digest MPs/NPs, lysosomal impairment, autophagy defect. A thorough analysis of the metabolic changes caused by exposure. MPs of distinct sizes pointed out different metabolomic changes, with 1139 upregulated and 815 downregulated metabolites in cells treated with the smaller MPs, compared with 1383 upregulated and 925 downregulated metabolites for larger counterparts. Pathways found altered by both treatments were those involved in arginine, proline, glutathione, and vitamin B6 metabolism. More importantly, 0.5 μm and, at a lesser degree, 5 μm MPs affected sphingolipid metabolism, with a significant reduction in sphingosine and 3-dehydrosphinganine.

Metabolic changes upon exposure to plastic MPs/NPs also occur in primary human monocytes. Alijagic et al. [10] used polyamide MPs, either pristine or reused, to treat monocyte-derived macrophages and investigated the behavior of 38 polar metabolites. While no significant cumulative difference was seen among the different sample groups, several single metabolites exhibited consistent changes. Interestingly, one of the altered pathways is related to tryptophan metabolism, with kynurenine increased upon exposure. Conversely, nicotinamide adenine dinucleotide (NAD) and flavin adenine dinucleotide (FAD) consistently increased in cells from each of the three enrolled donors but only upon exposure to reused polyamide MPs. Kynurenine, FAD, and NAD are components of the kynurenine metabolic pathway, which has an important immunoregulatory role. As underlined in that study, kynurenines can modulate aryl hydrocarbon receptor (AhR) activity, thus potentially regulating several inflammatory pathways [47].

## 7. MPs/NPs, Macrophages, and Microbial Communities

The interaction of nanomaterials with the human microbiota, i.e., the microbial communities residing in the human body, has elicited an increased interest since it could represent an indirect mechanism potentially accounting for the biological effects of the materials [48]. This possibility is particularly pertinent for materials whose preferential exposure route is represented by ingestion, as in the case of MPs/NPs, given the richness and complexity of gut microbiota. The current scientific literature focusing on this topic is still rather limited and somewhat contradictory but provides some interesting clues.

Mice exposed chronically to real-life compatible doses of PS MPs showed no weight loss or substantial changes in the microbiota composition at the genus, class, or phylum levels [49]. Also, Principal Component Analyses (PCA) and Shannon Diversity Indices showed no significant effect of PS MPs on the microbiota complexity. However, although the study did not document the presence of materials in internal organs, the treatment caused transcriptional changes, pointing to mild gut inflammation and, more importantly, a significantly prolonged inflammatory lesion (arthritic foot swelling induced by Chikunguya virus infection, associated with elevated immune cells). The authors suggest that this clinical picture may resemble human Inflammatory Bowel Disease (IBD)-associated enteropathic arthritis due to the activation of the colon’s innate lymphoid cells.

In contrast, changes in gut microbiota, depending on exposure to MPs, have been documented by other studies [1,50,51,52,53]. In particular, Schwenger et al. [50], assessing the role of MPs in the development of metabolic-associated fatty liver disease (MAFLD) and steatohepatitis (MASH), observed a positive correlation between fecal MP fibers and fecal *Bifidobacterium* and a negative correlation with members of the Lachnospiraceae taxon.

Somewhat similar modifications have been reported in a wild-world salamander by Li et al. [54]. Salamander larvae were exposed to different concentrations (0, 0.04, 0.2, 1, 5 mg/L) of PS-NPs (80 nm). Exposed animals presented increased hepatic macrophages with changes in antioxidant- and inflammation-related enzymes. In parallel, the relative abundance of *Cetobacterium* and *Akkermansia* decreased in the gut microbiota of these animals, while that of *Lachnoclostridium* increased. Besides its value in extending our knowledge on the conservation issue raised by MPs/NPs, this study has obvious implications for plastic-related pathogenesis of liver and gut conditions.

The involvement of MPs-/NPs-dependent gut microbiota alterations in extraintestinal conditions has been suggested by Kuai et al. [4], who attributed depressive changes in PS MPs-exposed mice to alterations of the microbiota–gut–brain axis. The survey of the gut microbiota composition evidenced that 44 operational taxonomic units (OTUs) were altered, with 7 genera identified as the most significantly altered. Remarkably, it has been profiled as an enhancement of the relative load of *Bacteroides*, *Lactobacillus*, and *Odoribacter* in the exposed animals, with a significant reduction in Parabacteroides.

The effects of PET NPs on the gut microbiota composition have been studied by Harusato et al. [46]. In the absence of clear-cut inflammatory changes, chronic, low-dose exposure of mice to PET NPs did not change the overall composition of the microbiota, although bacterial families normally present in human feces were found to be reduced (such as Atopobiaceae) or increased (Sutterellaceae). A different approach was adopted by Zhou et al. [55], who investigated the metabolic arsenal of the members of the gut microbiota for PET hydrolases, which are key enzymes involved in the degradation process of PET, finding at least five microbially encoded PET hydrolase candidates. The activity of these enzymes, expressed upon recombination in *Escherichia coli*, was tested on PET MPs powders and amorphous films. Mono(2-hydroxyethyl) terephthalic acid, terephthalic acid, and ethylene glycol were found released by four of the five enzyme preparations, with significantly higher activity on amorphous PET films, whose surfaces presented pits and grooves. The released compounds were able to modulate gene expression in murine macrophages in vitro, inducing M1 cytokines and inhibiting the expression of M2 cytokines.

## 8. Inflammation and Inflammation-Related Disease

MPs and NPs have been repeatedly implied in inflammatory reactions in exposed tissues given their widespread presence in organs and systems and the effects of these materials on macrophage polarization and activation. Specific mechanisms underlying the pro-inflammatory activities of MPs/NPs are being defined. Using the human macrophage-like THP-1 cells, Busch et al. [56] tested the pro-inflammatory activity of eight MPs/NPs of different sizes, shapes, and chemical compositions. While amino-modified polystyrene (PS-NH2) activated the nucleotide-binding oligomerization domain-like receptor family pyrin domain-containing 3 (NLRP3) inflammasome, the others were ineffective. However, PET, polyacrylonitrile (PAN), and nylon (PA6) promoted IL-8 secretion by NLRP3-/- cells, suggesting that some, but not all, MPs/NPs activate the NLRP3 inflammasome, while other materials are evidently able to trigger inflammasome-independent inflammatory responses. NLRP3 activation by PS MPs in THP-1 macrophages has also been investigated by Jeon et al. [57], who investigated particles of different sizes (1 µm vs. 10 µm), UV pre-treatment (pristine vs. UV-dependent oxidation), and origin (secondary vs. primary). Cytotoxicity and expression of inflammatory cytokines were dependent on size (1 µm > 10 µm), UV oxidation (UV > pristine), and origin (secondary > primary).

Also, the induction of chemokines seems dependent on the type of MPs/NPs considered. For instance, Kwabena Danso et al. [58] report increased levels of the chemokines MIP-1α (CCL3), MIP-2 (CXCL2), KC (CXCL1), and MCP-1 (CCL2) in the BALF of mice exposed to PS MPs/NPs. Instead, no MCP-1 increase was found by Alijagic et al. [10] in mice exposed to polyamide NP, while Woo et al. [59] report the increase in BALF KC and MCP-1 in mice exposed to PP MP/NP. From these data, it is not only evident that chemokine induction is influenced by the type of MC/NP but also that the effect involves chemokines preferentially active towards either monocytes or neutrophils.

### 8.1. Lungs and the Respiratory System

Since inhalation and ingestion are the most common exposure routes, it is not surprising that toxic effects on respiratory and gastrointestinal systems have been extensively investigated. Xia et al. [60] studied the acute (1 d and 3 d) effects of fluorescent PS MPs, instilled in the trachea, on the lungs, thymus, spleen, testicles, liver, kidneys, and brain. Exposed mice showed infiltration of blood cells, along with MPs presence, in most of these tissues, with a TNF-α and IL-1β increase in the lungs, thymus, spleen, and liver. These effects were mitigated by Toll-like receptors (TLR2 and TLR4) inhibitors, suggesting a somewhat specific recognition of materials by innate immune cells. However, no secretion of MCP-1 or TNF-α was detected by Raw264.7 cultures, suggesting that inflammatory response to MPs in vivo requires factors or conditions not always reproducible in vitro. It should be noted, however, that the in vitro experiments have been performed with 3 μm MPs, which were not internalized by the murine macrophages, possibly for a dimensional restriction.

Nasal instillation of PS MP was the exposure method adopted by Liu et al. [61]. Performing both in vitro and in vivo experiments, these authors found pulmonary fibrosis, necrosis, and excessive double-stranded DNA release into serum and BALF in C57BL/6 mice. These changes were indicative of strong oxidative stress, which was linked to cell death, inflammatory response, and the activation of the cyclic GMP-AMP synthase (cGAS) stimulator of interferon genes (STING)-signaling pathway described in Raw264.7 cells.

The possible differential inflammatory lung toxicity of MPs/NPs of different compositions has been investigated by Kwabena Danso et al. [58]. These authors compare the effects of sub-chronic (14d) instillation of PP, PS, and PE MPs on the respiratory system of mice. While inflammatory cells and cytokines increased in the BALF of PS-instilled mice, PP- and PE-exposed animals did not show significantly enhanced inflammatory changes. TLR4 was increased in the lung tissue of PS-treated animals, along with NF-κB phosphorylation and NLRP3 inflammasome components (ASC and Caspase-1).

The molecular mechanisms involved in lung damage are also starting to be defined. Sun et al. [62] used a chronic inhalation model to study the development of emphysema in rats exposed to PS MPs. Exposed animals showed inflammatory cell infiltration, septal thickening, alveolar dilatation, ECM degradation-related markers MMP9 and MMP12, and lowered elastin abundance in lung tissues. The expression of the circRNA_SMG6 decreased in both rat lung tissues and human THP-1 cells, while its overexpression mitigated PS-MPs-induced ECM degradation. Authors suggested that cRNA_SMG6 may regulate miR-570-3p and repress MMP enzymes blocking ECM degradation by macrophages. A different mechanism has been proposed for PP NPs. The exposure to this material would activate p38 kinase and the NF-κB pathway, causing mitochondrial damage, which would promote the lung changes detected after instillation in treated animals, such as parenchymal infiltration of inflammatory cells, alveolar epithelial hyperplasia, and foamy macrophage aggregates [59].

### 8.2. Gut

The increasing prevalence of IBD in the Western world has prompted research into the etiologic factors involved. The possibility that MPs/NPs are one of these factors has been investigated by Schwarzfischer et al. [63]. The pro-inflammatory effects of 25 nm polymethacrylate (PMMA) or 50 nm PS particles were readily observed in bone marrow-derived murine macrophages (BMDMs), from either morphological changes or pro-inflammatory gene induction. Conversely, mice exposed for six months to PMMA NPs before the induction of acute dextran sodium sulfate (DSS) colitis showed no exacerbation of the condition. This lack of effect, defined as “unexpected” by the authors, led them to conclude that IBD patients could be somehow reassured about the effects of NPs ingestion. However, the report does not present any data on the possible effects of NP of different compositions, such as PS, in the same model. Moreover, it is possible that the experimental procedure adopted, causing severe, acute colitis, masks the subtler effects of the nanomaterial. Exacerbation of DSS-induced colitis has instead been reported by Zolotova et al. [64] after a 6-week oral administration of 5 μm PS MPs. These authors report an increase in the number of endocrine cells, the content of highly sulfated mucins in goblet cells, and the number of cells in the lamina propria. While PS NPs do not further increase innate immunity cell infiltration (evaluated as the volume fraction of mucosal tissue attributed to these cells) in animals with colitis, macrophages significantly decrease in control animals exposed to NPs.

However, changes in the gut immune system dependent on exposure to plastic NPs do not necessarily require frank inflammatory changes, at least in the case of PET. Chronic, low, real-life exposure to PET NPs did not alter mucin barriers or the abundance of immune cells, including macrophages, but had a marked impact on the transcriptome and metabolism of gut CD45^+^ immune cells [46].

The effects of PS NPs on the gut–liver axis have been comprehensively investigated by Chen et al. [65]. After a 21 d exposure to PS-NPs, the material was found in the stomach, intestine, liver, lungs, kidney, brain, and testicles. An acute inflammatory reaction was evident from either histopathological analysis or blood tests, associated with the activation of NF-κB/NLRP3 pathways, the expression of cytokines (IL-1β and IL-18), and the accumulation of macrophages and neutrophils into the intestine. Interestingly, the intestinal permeability barrier was impaired with lowered expression of tight junction proteins (Claudin-1, Occludin, and ZO-1) and increased blood endotoxin levels. High LPS, in turn, activated TLR4/NF-κB/NLRP3/GSDMD pathways in the liver, with liver inflammation and hepatocyte pyroptosis, a form of regulated necrosis (see below, Section 8.3).

Persistent inflammation is now considered one of the hallmarks of cancer, and its role is important in some forms of tumors, such as colon cancer. Yang et al. [66] exploit the oral administration of PE and PS NPs to study their relationship with gut immunity and colon carcinogenesis. Through single-cell RNA sequencing, NPs were found to promote macrophage lysosomal damage and IL-1β-production, in turn causing Treg and Th17 differentiation and, more importantly, T cell exhaustion. Defective adaptive immunity is believed to favor tumor growth, as directly shown by the same authors for PE NPs in an orthotopic colon cancer model, where more immunosuppressive (F4/80^+^CD206^+^) tumor-associated macrophages (TAMs) were found [66].

### 8.3. Liver

Among the conditions related to inflammation, the progression from metabolic dysfunction associated with fatty liver disease (MAFLD, previously named NAFLD, non-alcoholic fatty liver disease) to steatohepatitis (MASH) is gaining particular attention, due to the increasing prevalence of these conditions. Assessing the role of MPs in the development of MASH, Schwenger et al. [50] noted no significant differences in fecal MPs between patients and healthy donors, but an association between fecal MPs fibers and total MPs with portal and total macrophages was found. Interestingly, when MASH patients were re-assessed after post-bariatric surgery, those with persistent disease had higher fecal MPs, suggesting that MPs can promote the progression of the inflammatory condition.

The hepatotoxicity and steatogenic potential of MPs have been recently documented in an advanced in vitro model [67]. PS MPs (1 and 5 μm) were incubated with a functionally active 3D liver model, including primary human hepatocytes, Kupffer cells, sinusoidal endothelial cells, and hepatic stellate cells. Increased cell death and inflammation, detected from the release of IL-6, IL-8, and TNF-α, were observed, along with a distortion of tissue organization and the presence of large lipid droplets inside the cells in the liver, especially Kupffer cells.

Altered liver lipid metabolism and the deranged gut–liver axis were reported also by Zhao et al. [68]. They supplied C57BL/6J mice with PS microbeads of two sizes (0.5 and 5 µm) for 13 weeks. Exposed mice gained more weight and adipose tissue, with an increased presence of activated F4/80^+^ macrophages. MAFLD was not detected, perhaps due to the short exposure time, but hepatic farnesoid X receptor and liver X receptor signaling were enhanced, with increased cholesterol and bile acids. Interestingly, delphinidin, a berry anthocyanin, mitigated weight gain.

Single-cell transcriptomics has favored the identification of the roles of MPs’/NPs’ effects on specific cell types in liver steatosis. Working on the zebrafish model, Deng et al. [69] defined what liver cell types are involved in PS NPs effects. Hepatocytes, macrophages, and lymphocytes are the most sensitive cell types, exerting specific and different transcriptional responses. Among immune cells, macrophages were most affected by the treatment (278 differentially expressed genes (DEG), 247 of which are only in macrophages). DEG shared by macrophages and lymphocytes concerns oxidative phosphorylation, while amino acid biosynthesis and metabolism, glycolysis, lipid metabolism, and intestinal immune network for IgA production were identified as the pathways of gene expression specifically affected in macrophages. Single-cell transcriptome analysis in a murine model of MAFLD. Ref. [70] indicated that the co-administration of PS MPs and a high-fat diet worsened the histopathological changes in the liver, such as hepatic steatosis, inflammatory cell infiltrations, and ballooning degeneration. Transcriptomic analysis indicated that MPs enhanced inflammation and the macrophage shift between different M2 polarized cells, with increased Vsig4+ and decreased angiogenic S100A6+ cells.

These results may suggest that liver damage evolution to fibrosis may be caused by defective resolution of acute damage, as suggested by Qian et al. [71]. These authors have investigated the chronic effects of PS MPs/NPs exposure on the classical model of acute liver injury by carbon tetrachloride. They found that PS exposure worsened acute liver injury, while *Gsdmd* knockout or inhibition by necrosulfonamide mitigated damage and inflammation, indicating the involvement of pyroptosis. This was corroborated by the demonstration in vitro of pyroptosis of Kupffer cells exposed to PS. The mechanisms underlying liver fibrosis by PS MPs have also been investigated by Wang et al. [72]. These authors adopted a prolonged protocol of administration of 30 d, showing macrophage recruitment and the ROS-independent formation of macrophage extracellular traps (METs), which are more evident with larger MPs. Macrophage traps induced epithelial–mesenchymal transition (EMT) of hepatocytes via activating the ROS/TGF-β/Smad2/3 signaling axis, a process mitigated by DNase I. PS MPs-induced MET formation has been studied by Yin et al. [73], who, however, attribute it to oxidative mitochondrial stress and disruption of mitochondrial homeostasis, with autophagy activation, eventual lysosome rupture, and the release of calcium ions. Antioxidants and autophagy inhibitors mitigated the damage.

Abnormal crosstalk between hepatocytes and liver macrophages is considered of pivotal importance in the pathogenesis of liver damage by MPs and NPs [74]. In this study, 20 nm synthetic PS NPs, but not larger particles, caused macrophage death with features of necroptosis, a type of regulated, pro-inflammatory, necrotic cell death. Authors attributed the process to mitochondrial accumulation of the materials and enhanced production of ROS. Possibly through the triggering of inflammation, macrophage necroptosis hindered hepatocytes, leading to liver damage that was prevented by a necroptosis inhibitor or by depletion of macrophages. However, it should be considered that liver toxicity by MPs/NPs is not limited to PS. Boran et al., using a HepG2/THP-1 co-culture model, demonstrated that, also, MPs of PMMA (polymethyl methacrylate) cause lipid accumulation, oxidative stress, and inflammatory changes [75].

### 8.4. Bone

Our group [76] investigated the effects of PS NPs on three murine cell lines (MC3T3-E1 preosteoblasts, MLOY-4 osteocyte-like cells, and Raw264.7 macrophages that can be induced to differentiate into osteoclasts). In all three lines, NPs lower cell viability and induce ROS production and apoptosis. Significant promotion of osteoclastogenesis was also detected from the expression of inflammatory and osteoblastogenic genes linked to osteoclastogenic commitment. Moreover, osteoblastogenesis was impaired, further altering the bone remodeling balance.

### 8.5. Cardiovascular System

The atherogenic potential of exposure to plastic MPs/NPs has summoned increasing interest, and a significant role is played by inflammation and alterations of lipid metabolism (see above, inability to digest MPs/NPs, lysosomal impairment, autophagy defect). In this context, particular attention should be devoted to the coexistence of other risk factors. For example, the report by Wen et al. [77] includes in vitro experiments on the Raw264.7 model, showing that co-exposure of PS NPs with oxidized LDL leads to lipid accumulation, oxidative stress and pro-inflammatory activation. These data are complemented by experiments on a susceptible animal model, the ApoE-/- mouse, in which the development of atherosclerotic plaques is followed with an ultra-high-resolution color Doppler ultrasound system during the chronic (3 months) administration of PS NPs. Plaque formation is associated with alterations in liver lipid metabolism (see above, Liver).

### 8.6. Other Organs and Systems

Inflammation due to M1-type activation may be the mechanism underlying other effects attributed to the exposure to MPs, not directly related to the administration route. One of these effects is the testicular inflammation [45] with reduced male fertility [42]. Exposure to PS NPs causes vacuolization of seminiferous tubules with the presence of M1 macrophages, activation of macrophagic ROS production, atrophy of testicular cells and profound metabolic changes in the tissue [45]. In the study by Jin et al. [42], mice were orally exposed to PS-MPs for 180 days, causing the accumulation of M1 macrophages in the testicles. The consequent secretion of TNFα activated NF-κB in Leydig cells with the repression of the gonadotropin receptor LHR and the suppression of testosterone secretion.

Other effects attributed to exposure to plastic MPs/NPs consist of cognitive and metabolic defects. Yang et al. [78] synthesize PE MPs (~500 nm, ~2 μm) and expose mice to their food. As expected, smaller MPs (which authors define as NPs) activate gut macrophages better than MPs, leading to increased IL-1β production through lysosomal damage. Authors attribute microglial activation, Th17 differentiation, declining cognitive activity, and short-term memory to this effect. Brain penetration and microglial activation by PS MPs have also been described by Lee et al. [79], who demonstrated the widespread presence in the brain and microglial activation in the hypothalamus by PS MPs after oral administration. Through molecular docking experiments, the same authors demonstrate in silico that, among PE, PP, and PS, PS MPs have the highest binding affinity for macrophages, and that, upon oral administration to high-fat diet-induced obese mice, MPs are found adsorbed to immune cells in the blood and exacerbate impaired glucose metabolism, insulin resistance, and systemic inflammation.

Xu et al. [80] performed a single-cell transcriptomic analysis of the kidney microenvironment in mice orally exposed to PS MPs and a high-fat diet for 35 days. They found the worsening of the kidney profibrotic microenvironment, the activation of the PI3K-Akt and MAPK pathways, and the increase in CD8+ effector T cells. More M2-like PF4+ macrophages were detected, a finding to consider in the context of the increased presence of this type of cell in clear cell renal carcinoma and adjacent normal tissues, suggesting a role for PS MPs in renal carcinogenesis. In a different model, based on ischemia–reperfusion, Kuang et al. also studied the kidney toxicity of PS MPs [81]. Chronic (12 weeks) exposure to MPs worsened damage from ischemia–reperfusion to tubules and glomeruli and increased the expression of MCP-1 and IL-6 mRNAs, together with that of the macrophage markers CD68 and F4/80 and pyroptosis-related parameters (*NLRP3*, *ASC*, and *GSDMD* genes; cleaved caspase-1 and IL-18).

## 9. Conclusions and Open Issues

**Figure 1:**
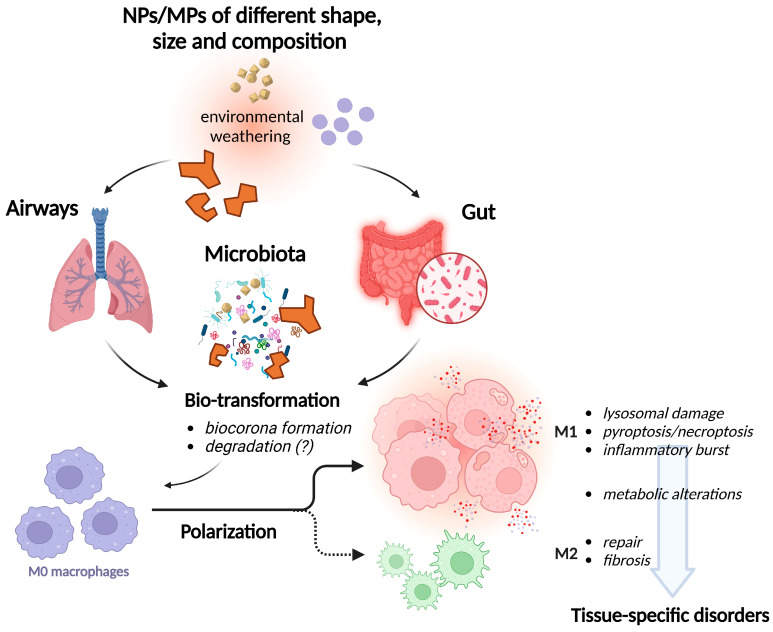
The main steps of the interaction of MPs/NPs with macrophages. The figure represents a unitary vision of the interaction of MPs/NPs with macrophages, from changes occurring before the exposure (environmental weathering) to organ disease, as the result of alterations of macrophages at the cell level. For polarization, the solid line represents the favored drive towards M1 polarization, while the dotted line represents the less common drive towards M2 polarization. See text for a more detailed description. Created in BioRender. Bianchi, M. n80u669.

The exceptional progress obtained in the last few years in the knowledge of macrophage biology and its role in human disease should allow the comprehension of multi-organ pathogenetic mechanisms elicited by MPs/NPs exposure (see Figure 1) and, hence, the implementation of preventive and therapeutic approaches. On the other hand, the awareness of possible health problems linked to the ever-increasing presence of MPs and NPs in the environment is the basis of the explosion of studies on this subject. The exceptional growth of the scientific literature published in these last few years has stressed the urgency to address important, so-far-unresolved issues.

Among these, there is a need to better understand composition-specific effects. This item requires additional research, given the great variability of plastic materials used. When directly compared under the same experimental conditions, PS MPs/NPs are more toxic than PP- and PE-based counterparts, at least as far as inflammatory changes in the lung are concerned [82]. These authors used several types of domestic waste plastic, obtaining NPs made of PET, PS, PP, high-density PET, and poly (ethylene-co-methacrylic acid). The exposure to NPs led to differential alterations in the expression of inflammatory genes in lung tissues, with a much higher expression of inflammatory cytokines and TLR4 in animals treated with PS MPs/NPs. Whether this hierarchy is true for pathological changes in other tissues and the mechanisms accounting for different toxicity remain to be established.

In real life, at variance with other types of nanomaterials, most MPs and NPs are secondary, i.e., derived from the disposal of common plastic goods. The increasing use of these secondary materials in studies on biological effects is to be commended since they adhere more strictly to real-life exposure scenarios. However, it renders necessary to apply specific assays to determine the chemical composition of the materials with methods such as Raman or FTIR spectroscopy, since they consist of various compounds, such as, for example, PET, PS, PP, and PE [83].

Another connected issue concerns the possibility of identifying and quantifying MPs and NPs in tissues or cells. A possible solution consists of doping the materials with metals (see, for instance, Cassano et al. [84], Zhang et al. [85]), or fluorescent dyes [86], so as to render them readily detectable and quantifiable. However, although technically demanding, detection of label-free MPs/NPs should be preferable [86], since doping may alter the properties of the materials, thus modifying their interactions with tissues or cells. Moreover, label-free techniques allow the detection of internalized MPs/NPs of environmental origin.

Although most experimental studies have been performed until now with pristine materials, the use of secondary materials in biological experiments is a prominent issue in terms of adherence to real-life situations and confers larger robustness to the findings. Indeed, secondary materials seem much more biologically active than pristine counterparts, due to the physicochemical modifications occurring during the persistence in the environment. For instance, Liu et al. [87] demonstrate that photoaging of tire wear particles leads to the formation of “environmentally persistent free radicals” that account for oxidative stress and viability decrease in THP-1 macrophages. The direct comparison performed by Vokl et al. [88] demonstrates that the response of murine macrophages to pristine and weathered MPs is qualitatively different, with a much larger transcriptional response, along with necrosis and viability loss, after exposure to extensively aged MPs. Quite paradoxically, a higher inflammatory activation, detected from TNF-α secretion, is observed after exposure to pristine material, although authors do not exclude an artifact due to the higher cytotoxicity of aged MP. Moreover, during exposure to environmental agents, it is likely that plastic micro-plastics further break down into nanosized fragments, potentially persisting in the environment for extended periods. This consideration should particularly prompt the study of the chronic effects of exposure to MPs and NPs.

In contrast, another study, where aged and pristine PS MPs are directly compared [89], indicated higher DNA damage and larger induction of IL-6 and NOS2 expression at mRNA level in Raw264.7 macrophages exposed to aged MP, together with reduced cell viability and increased ROS production. Consistent results have been obtained with human THP-1 macrophages by Visalli et al. comparing pristine and oxidized PS MPs/NPs [90]. The marked difference detected in the biological activity of pristine and aged MPs/NPs explains why methods have been published to “age” the materials under controlled conditions [83,89,90].

One of the reasons that render weathered MPs/NPs more active than pristine counterparts is the possibility that, during the period spent in the environment, the particles adsorb other materials. Zhao et al. [91] doped PS MPs with Cd and described enhanced toxic effects, such as lipid peroxidation in red blood cells and macrophages. Also, the increased formation of a protein corona during the aging process [28] can enhance the phagocytosis of MPs/NPs [26] and, hence, their biological effects.

A crucial area of investigation will also consist of studies aimed at better understanding the molecular interactions between MPs/NPs and the human microbiota, with potential consequences for the host. In fact, there are still open questions about the possible negative effects driven by MPs/NPs on various members of the human microbiota, which might lower the diversity of the complexity of microbial communities, i.e., of the dysbiosis effect. Dysbiosis is frequently associated with alterations of innate immunity and macrophage polarization and, hence, with inflammatory disorders [92,93]. Another intriguing issue is represented by the identification of naturally microbial MPs and NPs degraders, especially in geographical areas characterized by high plastic pollution [94], and by the possibility that degradation products also have biological effects.

In conclusion, since pollution by MPs and NPs is expected to increase further in the next few years independently of regulatory evolution [3], efforts for a comprehensive awareness of their effects on human health should be adequately promoted.

## Data Availability

No new data were created or analyzed in this study; therefore, data sharing is not applicable.

## Abbreviations

The following abbreviations are used in this manuscript:

BALF	Bronchoalveolar fluid
IBD	Inflammatory Bowel Disease
MAFLD	Metabolic dysfunction-associated fatty liver disease
MASH	Metabolic dysfunction-associated steatohepatitis
MPs	Plastic microparticles
NP	Plastic nanoparticles
PE	Polyethylene
PET	Polyethylene terephthalate
PP	Polypropylene
PS	Polystyrene
PVC	Polyvinylchloride
ROSs	Reactive oxygen substances
